# A Study Examining the Usefulness of a New Measure of Research Engagement

**DOI:** 10.1007/s11606-021-06993-1

**Published:** 2022-03-29

**Authors:** Deborah J. Bowen, Nicole Ackermann, Vetta Saunders Thompson, Andrea Nederveld, Melody Goodman

**Affiliations:** 1 Department of Bioethics and Humanities, University of Washington, Seattle, WA USA; 2 Division of Public Health Sciences, Washington University School of Medicine, St. Louis, MO USA; 3 Brown School, Washington University in St. Louis, St. Louis, MO USA; 4 Department of Family Medicine, University of Colorado Anschutz Medical Campus, Aurora, CO USA; 5 Department of Biostatistics, New York University School of Global Public Health, New York, NY USA

**Keywords:** participant engagement, research engagement, survey implementation, implementation science, stakeholder-engaged research

## Abstract

**Introduction:**

Engagement of relevant stakeholders’ ideas, opinions, and concerns is critical to the success of modern research projects. We have developed a tool to measure stakeholder engagement, called the Research Engagement Survey Tool (REST). The purpose of this paper is to present the implementation and uptake of the stakeholder engagement measure REST among research teams, including the assessment of barriers and facilitating factors for use of the new research engagement measure in practice.

**Methods:**

In this implementation study, project team members participated in baseline and follow-up web-based surveys. Web-based interviews were conducted with a subset of project teams that implemented the REST. On the baseline survey, project teams were asked to provide details about up to three ongoing or recently completed projects, were asked if they agreed with compensation for REST completion, and were asked if they would like to send the survey to stakeholders or would prefer our project team to email their project stakeholders. Follow-up surveys contained questions on reactions to implementing REST and results of REST.

**Results:**

Project team members/researchers who completed the baseline survey (n=86) were mostly female (79%) and Non-Hispanic/Latino(a) White (76%). Those who implemented REST were also mostly female (86%) and Non-Hispanic/Latino(a) White (71%), with an average of 11 years in academic research. About 98% of all participants completing the baseline survey had the capacity to survey partners, while 100% of all teams who implemented REST did. A small portion of respondents indicated the time commitment of REST would be a barrier (29% of baseline survey respondents, 10% of those who implemented REST) and indicated workload would be a barrier (31% of baseline survey respondents, 14% of those who implemented REST).

**Discussion:**

The data presented here indicate that REST implementation is feasible in a volunteer group of ongoing research projects.

**Supplementary Information:**

The online version contains supplementary material available at 10.1007/s11606-021-06993-1.

## INTRODUCTION

Engagement of relevant stakeholders’ (e.g., patients and their families, clinicians, health systems, policy makers, community organizations, advocacy groups) ideas, opinions, and concerns is critical to the success of modern research projects. To facilitate acceptance of the important role that stakeholder engagement plays in rigorous science, we must evaluate its impact on research development, implementation, and outcomes [1]. We have developed a tool to measure stakeholder engagement, called the Research Engagement Survey Tool (REST) [2, 3]. The utilization of REST as an evaluation tool depends on its dissemination into the hands of research teams and incorporation in the evaluation procedures of projects that might benefit from its use.

The comprehensive (32-item) version of the Research Engagement Survey Tool (REST) examines eight engagement principles (EPs) [2, 3], based on the stakeholder engagement literature [4–7]. The EPs are the following:

1. Focus on community perspectives and determinants of health.
2. Partner input is vital.
3. Partnership sustainability to meet goals and objectives.
4. Foster co-learning, capacity building, and co-benefit for all partners.
5. Build on strengths and resources within the community or patient population.
6. Facilitate collaborative, equitable partnerships.
7. Involve all partners in the dissemination process.
8. Build and maintain trust in the partnership.

Each EP is assessed using three to five items that are measured on two scales: quality (how well) and quantity (how often) using Likert response options. The REST was developed and validated using stakeholder-engaged approaches in collaboration with community advisory boards (Disparities Elimination Advisory Committee, Patient Research Advisory Board) and research methods to obtain feedback from key informants (e.g., Delphi process and cognitive response interviews) [2, 3, 8–10].

The REST is a valid and reliable quantitative survey tool to assess research engagement of non-academic stakeholders in the research process [2, 9]. Project teams can use this information to examine engagement strategies and the level of engagement of a project. Valid standardized tools (e.g., REST) that assess research engagement among non-academic stakeholders are necessary to examine the impact of stakeholder engagement on the scientific process and scientific discovery and move the field of stakeholder engagement from best practices and lessons learned to evidence-based stakeholder engagement approaches based on empirical data. The REST can be used to compare engagement across projects or within a single project over time.

Implementation is appropriately moving a research product to actual practice [11]. Knowledge implementation ensures that knowledge is available to those who need it [12]. Ideally, this dissemination focuses on “getting the right knowledge to the right people, in the right place at the right time” [12]. This suggests that simply publishing the development process is likely not enough to ensure that the tool receives wide usage in multiple projects.

Our approach to this pilot implementation study was influenced by theories that discuss factors affecting adoption and implementation of new scientific knowledge and tools. The literature suggests that dissemination activities should be planned with a focus on the needs of the stakeholders who will use the new tool [11]. In addition, it is important that tool developers engage potential users to better understand how to develop messages and disseminate findings and tools. Finally, knowledge brokers, networks, and those who will actually use the new tool, in this instance funders, research networks, and stakeholder-engaged researchers, are best positioned to help identify facilitators and barriers to the use of new knowledge and tools. In summary, successful dissemination requires active collaboration and exchange between tool developers and users throughout the research process.

Based on recommended activities of the dissemination literature, we decided to take an active approach to encouraging REST use. We proactively contacted multiple research teams around the country to consider using the tool in their evaluations and monitored the responses to the efforts to encourage use. The purpose of this paper is to present the implementation and uptake of the research engagement measure (REST) among research teams, including the assessment of barriers and facilitating factors for use of the new research engagement measure in practice.

## METHODS

### Participant Recruitment

To identify principal investigators of stakeholder-engaged studies, the study used the Patient-Centered Outcomes Research Institute (PCORI) website, National Institutes of Health (NIH) RePORTER, and a database of community engaged researchers developed during recruitment in previous research [2, 3, 9, 10]. Inclusion criteria for the recruitment of project teams include (1) teams with ongoing or recently completed (within the last calendar year) stakeholder-engaged projects, and (2) interest in working with this PCORI project to provide feedback on implementing the Research Engagement Survey Tool (REST), and, possibly, to implement it in a current or recently completed project. Additionally, during data collection, we added a screening question to determine if a tool available only in English would be useful to teams. If a tool in English only was not useful to teams, we deemed those teams ineligible for the implementation study. Forty-one Patient-Centered Outcomes Research/Community Engaged Research (PCOR/CEnR) project teams responded to the request to examine uptake among research teams, barriers, and facilitating factors for use of the research engagement measure.

Emails requesting participation were sent to a list of all publicly identifiable NIH Clinical and Translational Science Awardees community engaged, PCORI patient-engaged research teams, and the database of community-engaged researchers developed during recruitment in previous research mentioned previously. We sent 482 emails to principal investigators (some of whom had retired or moved to other institutions, some of the email addresses were no longer valid). We tracked the number of teams that responded via email, those who expressed interest in the project but had questions, as well as those interested in follow-up calls or emails and the results of this follow-up, the teams that declined participation, and the teams that did not respond. We sent follow-up emails to project investigators who did not respond. Principal Investigators, project managers, and engagement leads who worked on stakeholder engagement and/or other project team staff initiated enrollment in the study; 3 were excluded due to not having use for a tool available in English only, 114 enrolled in the study (completed consent), and 86 completed baseline surveys were received (see Fig. 1 in the Supplementary Information); these include responses from more than one member of the same project team. We were not aware of the composition of the PCOR/CEnR teams and relied on the investigators contacted to make appropriate referrals but monitored the number of team members completing the survey and their role on the project. Because we were not aware of the size of the staff who were eligible to participate in the survey, we do not know how many may have declined in this effort. Teams were separate groups of investigators and staff focused on a study or studies, and stakeholders were the non-academic individuals who comprised each team (i.e., community advisory board members, patients, healthcare organizations). Recruitment of project teams started in September 2019 and continued through July 2020, though the last team to complete a baseline survey and go on to implement REST was recruited in April 2020.

### Procedures

The institutional review boards at Washington University in St. Louis and at New York University approved this study and the consent procedures used. Project team members received a $20 gift card for completing each web survey (baseline and follow-up), and a $40 gift card for completing the interview. They were also entered into a raffle for a tablet if they completed both the baseline and follow up web surveys.

After recruitment of the majority of project teams and completion of baseline surveys, teams who indicated they were interested in implementing REST were contacted via email with information on the next steps of implementing REST in their project (n=41 teams, 47 baseline survey participants). On the baseline survey, project teams were asked to provide details about up to three ongoing or recently completed projects, were asked if they agreed with compensation of stakeholders, and were asked if they would like to send the survey to stakeholders or would prefer our project team to email their stakeholders. Based on responses to these questions, we confirmed with teams which project(s) they would like to implement REST in, confirmed a project name stakeholders would recognize to use on the survey, and confirmed type of gift card or offered additional options for stakeholder compensation to teams. Of the 41 teams emailed next-step information, 20 teams continued on to implement REST, leaving a total of 21 teams who did not implement REST (see Fig. 1 in the Supplementary Information for details).

For teams that indicated they would like us to send the survey to their stakeholders, we also requested a contact list with stakeholders’ names and email addresses, then created a project specific link and sent to stakeholders. For teams who indicated they would like to send the survey to their own stakeholders, we provided a project-specific link to the survey for the team to distribute to stakeholders. The survey sent to project team stakeholders included the comprehensive 32-item version of REST, demographic questions, and a final question asking stakeholders to indicate where they believe that their projects fell along a continuum of engaged research, after being provided the definitions of categories of stakeholder engagement in research^10^. The five categories are defined based on engagement activities that represent nonacademic stakeholder activities and interactions with academic researchers. The continuum begins with none to limited stakeholder inclusion and input into research and continues with descriptions of increasing presence, input, and participation in decision-making. The categories and definitions used were subjected to review using Delphi process, and cognitive response interviews^9,10^. During implementation of REST, up to two email updates were provided to project teams on how many stakeholders had completed the survey. Stakeholders received a $20 gift card for completion of the survey.

Finally, after implementation of REST was complete, we returned results to project teams, along with an invitation to complete a follow-up web survey and a qualitative Zoom interview to provide feedback on the process of implementing REST and the results they received. Project teams indicated what form of results they would like to receive on the baseline survey. Teams could choose any or all of the following: a detailed project specific report, a detailed comparison report comparing their project to other projects in the study, and/or raw de-identified project specific data with specified data type (i.e., Excel, SAS, Stata). We developed these materials for each team who had at least five stakeholders complete the survey. Due to issues with data no longer being anonymous in samples smaller than five, projects who had less than five stakeholders complete the survey were instead provided a detailed report of overall data across all projects. An example of the report sent to project teams is available on the REST website (https://tinyurl.com/RESTtool). To complement the reports and data sent to project teams, we created a video going over the reports and data that was approximately 18 min long. This video is available on the REST website.

### Survey Instruments

Project team members were asked to complete a baseline survey and those that implemented the REST were asked to complete a follow-up survey after implementation. The baseline project team survey collected demographic information of the project team member completing the survey (gender, race, ethnicity, location, years in academic research) and information on project teams to help guide implementation of REST. We also collected feedback on perceptions of the barriers to implementing REST and potential feasibility, information surrounding current efforts of engagement and measuring engagement, communication with stakeholders, and interest in working with us to implement REST. We calculated frequencies and percentages of survey questions.

For the project team follow-up web survey, frequencies and percentages of items were calculated. Follow-up surveys contained importance of measuring stakeholder engagement in research, likelihood of recommending REST to a colleague, other tools used to measure engagement, feasibility of implementing REST, usefulness of REST informational video, able to understand results provided in reports, agree with project classification, amount of time and number of people involved in implementing REST and discussing results, confidence in ability to implement findings from REST, willingness to participate in follow-up interview, and questions surrounding how the COVID-19 pandemic impacted teams and partnerships.

For the project stakeholder survey, overall REST quality and quantity scores and scores by engagement principle were calculated for all teams and by project for those projects requesting project-specific data and had more than five stakeholders completing. We also calculated frequencies and percentages of responses for each item for both scales (quality and quantity) and for project classification by category of engagement (outreach and education, consultation, cooperation, collaboration, partnership).

## RESULTS

Figure 1 in the Supplementary Information presents the loss to follow-up at each survey stage of the implementation survey efforts. As seen in Fig. 1 in the Supplementary Information, only about half of the project teams approached agreed to implement REST, and of those, only half actually implemented REST in their projects.

Table 1 displays the demographic data associated with project team members that completed the baseline survey compared to projects that implemented REST as part of their ongoing work. Project team members/researchers who completed the baseline survey (n=86) were mostly female (79%) and Non-Hispanic/Latino(a) White (76%). Those who implemented REST were also mostly female (86%) and Non-Hispanic/Latino(a) White (71%), with a mean of 11 years in academic research (Table 1). Table 2 shows demographics of the project teams’ stakeholders who completed the REST survey. Stakeholders were mostly female (73%), Non-Hispanic/Latino(a) White (66%), with a graduate degree (56%) and mean age of 50.

**Table 1:** Demographics of Project Team Baseline Survey Participants—All Participants and Those Implementing REST

Characteristic	Category	All completions (N=86)	Implemented REST (N=21^1^)
		N (%)	
Race	Non-Hispanic/Latino(a) Black	9 (10.5%)	1 (4.8%)
	Non-Hispanic/Latino(a) White	65 (75.6%)	15 (71.4%)
	Hispanic	4 (4.7%)	3 (14.3%)
	Asian	8 (9.3%)	2 (9.5%)
	Other/ Multiracial/ Unknown	0 (0%)	0 (0%)
Gender	Male	17 (19.8%)	3 (14.3%)
	Female	68 (79.1%)	18 (85.7%)
	Other	1 (1.2%)	0 (0%)
Region	Midwest	21 (24.4%)	5 (23.8%)
	North East	25 (29.1%)	5 (23.8%)
	South	19 (22.1%)	5 (23.8%)
	West	21 (24.4%)	6 (28.6%)
Mean (SD)			
Years in Academic Research Environment		11.4 (7.4)	10.9 (7.3)

^1^One team implementing REST had more than one project team member complete baseline survey

**Table 2:** Demographics of Stakeholders Who Completed the REST Survey (n=173). One Hundred Seventy-Three Stakeholders Completed the REST Implementation Survey; However, One Stakeholder Was Involved in Two Projects and Two Stakeholders Were Involved in Three Projects, for a Total of 178 Survey Completions Across the 26 Projects

Characteristic	Category	N (%)
Race	Non-Hispanic/Latino(a) Black	34 (19.7%)
	Non-Hispanic/Latino(a) White	114 (65.9%)
	Hispanic	19 (11.0%)
	Asian	4 (2.3%)
	Other/multiracial/unknown	2 (1.2%)
Gender	Male	46 (26.6%)
	Female	127 (73.4%)
Education	< HS, HS degree or GED	9 (5.2%)
	Some college or associate degree	27 (15.6%)
	College degree	41 (23.7%)
	Graduate degree	96 (55.5%)
Region	Midwest	32 (18.5%)
	North East	30 (17.3%)
	South	32 (18.5%)
	West	77 (44.5%)
	Caribbean	2 (1.2%)
Mean (SD)		
Age (n=172)		50.1 (13.9)

We worked with 20 teams to implement REST in 26 projects. Five teams implemented REST in more than one project: four teams implemented REST in two projects, while one team implemented REST in three projects. Of the 20 teams that implemented REST, six teams had our research team email their stakeholders the survey containing REST (27% of stakeholder survey responses), while the remaining 14 teams sent the survey to their stakeholders themselves (67% of stakeholder survey responses). One team who implemented REST in three of their projects initially had our team email their stakeholders; however, towards the end of REST survey implementation, for one of their projects, they included additional stakeholders they emailed (6% of stakeholder survey responses). In half of the projects (n=13, 50%), the main contact who filled out the project team survey was the principal investigator (PI). The main contact was the project manager/coordinator for 9 projects (35%) and the remaining four projects, the contact was either a community/stakeholder engagement lead, co-PI, engagement project manager, or research director. Projects included a variety of types of stakeholders, including but not limited to community advisory board (n=14, 54% of projects), patients (n=19, 73%), healthcare organizations (n=20, 77%), study participants (n=12, 46%), community members (n=19, 73%), local community organizations (n=16, 62%), and health departments (n=10, 39%). Details of the projects that implemented the REST are presented in Table 3.

**Table 3:** Details of Projects in Which REST Was Implemented (n=26 Projects). Note: There Was One Project in Which REST Was Implemented That Was Not Captured on the Baseline Survey; Thus, Project Information Was Collected on the Follow-up Survey and One Project in Which the PI and Research Assistant Completed the Survey and the Answers Were Similar So Only the PI Responses Are Shown

Characteristic	Category	N (%)	
Project role	Principal investigator	13 (50%)	
	Community/stakeholder engagement lead	1 (3.9%)	
	Project manager/coordinator	9 (34.6%)	
	Other^1^	3 (11.5%)	
Type of stakeholders^2^	Community Advisory Board (CAB)	14 (53.8%)	
	Patients	19 (73.1%)	
	Healthcare organizations	20 (76.9%)	
	Study participants	12 (46.2%)	
	Community members	19 (73.1%)	
	Local community organizations	16 (61.5%)	
	Health departments	10 (38.5%)	
	Other^3^	11 (42.3%)	
Years stakeholders engaged	Less than 1 year	2 (7.8%)	
	1–2 years	9 (34.6%)	
	3–5 years	11 (42.3%)	
	Over 5 years (*max*=10)	4 (15.4%)	
Years worked on project	Less than 1 year	3 (11.5%)	
	1–2 years	13 (50.0%)	
	3–5 years	9 (34.6%)	
	Over 5 years (*max*=7)	1 (3.9%)	
	Mean (SD)	Range	Sum
Estimated number of stakeholders—individuals	40.3 (43.9)	3–150	1,048
Estimated number of stakeholders—groups	11.1 (11.0)	2–50	288
Actual number of stakeholders—individuals^4^	6.8 (6.3)	1–26	178

^1^Includes co-investigators, engagement project manager, and research director

^2^Check all that apply field so % out of 26 will not add up to 100

^3^Includes clinicians, payers/healthcare plans, social workers, national healthcare organizations, stakeholder advisory committee, university agricultural education center, self-advocates

^4^This is calculated from actual number of stakeholder survey completions per project

About 58% of projects had engaged stakeholders in their project for three or more years, while approximately 38% of project team members had worked on the project for three or more years, indicating that there were some projects in which stakeholders appeared to be involved for a longer amount of time than that participant project team member representing the project. On the baseline survey, project teams estimated an average of 40 stakeholders involved per project, with a range of 3 to 150 individuals. Project teams estimated an average of 11 stakeholder groups, with a range of 2 to 50 groups per project. The total estimated number of individual stakeholders involved in all projects that REST was implemented was 1,048. Actual number of stakeholders completing the REST survey was much lower than estimated. An average of seven stakeholders per project completed the REST survey, with a total of 178 survey completions across all 26 projects. By project, this corresponded to an average response rate of 31% per project, with a range from 0.1 to 88% based on the number of stakeholders estimated in the baseline survey. When stratified by method of survey distribution, the average response rate was 35% for those projects (n=7 projects) in which we emailed their stakeholders the survey (range: 0.1 to 67%) and the average response rate was 29% for those projects (n=18 projects) in which project team members emailed their own stakeholders the survey (range: 0.1 to 88%). The one project described earlier that included a mixture of our team emailing stakeholders and the project team emailing stakeholders had a response rate of 33% based on the number of stakeholders estimated in the baseline survey.

On the baseline survey, we also asked several questions about participants’ perceptions of the barriers to and feasibility of implementing REST and questions related to current capacity to survey their stakeholders. This information is presented in Table 4. About 98% of all participants completing the baseline survey had the capacity to survey stakeholders, while 100% of all teams who implemented did. Only approximately 36% of baseline respondents currently administered evaluation/satisfaction surveys to stakeholders, compared to 43% of those who implemented REST. A small portion of respondents indicated the time commitment of PI or staff would be a barrier to REST implementation (29% of baseline respondents, 10% of those who implemented REST) and indicated workload would be a barrier (31% of baseline respondents, 14% of those who implemented REST). On average, teams reported that about 93% of stakeholders (average of 95% of stakeholders for those implementing REST) had regular access to the Internet (indicating feasibility of a web-survey).

**Table 4:** Project Team Baseline Survey—Barriers and Feasibility of Implementing REST

Variable	Category	All completions (n=86)		Implementing only (N=21)	
		N (%)		N (%)	
Have capacity to survey stakeholders	Yes	84 (97.7%)		21 (100%)	
	No	2 (2.3%)		0	
Currently administer stakeholder evaluation/satisfaction survey	Yes	31 (36.1%)		9 (42.9%)	
	No	55 (63.9%)		12 (57.1%)	
Ever sent web survey^1^	Yes	22 (71.0%)		8 (88.9%)	
	No	9 (29.0%)		1 (11.1%)	
Barrier of PI/Staff Time Commitment	Yes	24 (28.6%)		2 (9.5%)	
	No	60 (71.4%)		19 (90.5%)	
Barrier of PI/staff workload	Yes	26 (31.0%)		3 (14.3%)	
	No	58 (69.1%)		18 (85.7%)	
Prefer to send the engagement survey to stakeholders	Study investigators	15 (18.3%)		4 (19.1%)	
	Their own project team	58 (70.7%)		16 (76.2%)	
	Other^2^	9 (11.0%)		1 (4.8%)	
		Mean (SD)	Range	Mean (SD)	Range
Percentage of stakeholders that have regular internet access		93.0 (14.3)	12–100	95.2 (9.8)	60–100
Percentage of people that participate^1^ (n=30, n=8)		67.2 (27.2)	5–100	68.8 (24.3)	20–100
Feasibility of intervention measure^3^					
The Research Engagement Survey Tool (REST) seems implementable.		3.8 (0.8)	2–5	4.3 (0.6)	3–5
The REST seems possible.		3.9 (0.7)	2–5	4.3 (0.5)	4–5
The REST seems doable.		3.8 (0.8)	2–5	4.3 (0.5)	4–5
The REST seems easy to use.		3.6 (0.8)	2–5	4.1 (0.6)	3–5

^1^Asked only of those who currently administer surveys

^2^Responded that either is fine or unsure

^3^Scale ranges from 1 (completely disagree) to 5 (completely agree)

Of the 20 teams that implemented REST, 13 (65%) requested results back in all three forms: project specific report, comparison report, and raw de-identified project specific data. Two teams (10%) requested only a comparison report, two teams (10%) requested only a project specific report, two teams (10%) requested raw de-identified project specific data and a project specific report, and one team (5%) requested both a project specific report and a comparison report. Because only 12 teams (with 14 total projects) had more than five stakeholders completing the REST implementation survey, we were not able to provide requested results for eight of the teams. After accounting for this, we were able to provide nine teams with all three form of results as requested, two teams with raw de-identified project specific data and a project specific report as requested, and one team with a comparison report as requested.

On the follow-up survey (Table 5), we asked questions of each team who implemented REST (n=20) and also asked questions specific to each project (n=26); thus, some teams answered project-specific questions about more than one project. We found that most teams were likely to recommend using REST to a colleague (median: 8.0 on a scale of 0 [not at all likely] to 10 [extremely likely]; range: 6 to 10). Only 45% (n=9) of teams reported watching the information video provided, while eight of the nine teams who watched (89%) reported finding the video useful. All but one team (90%) reported being able to understand the results they were given on REST; one team (5%) did not respond to this question. Because projects were only given project-specific results if they had five or more stakeholders complete the survey, we were only able to ask thoughts on how their project was classified for 14 of the 26 projects. Of those 14 projects who received project specific results, 12 projects (86%) reported they agreed with their project classification; one project (7%) did not respond to this question. Here classification is “level of engagement” based on how stakeholder partners responded to the item that asked them to classify their project on the research engagement continuum in one of the five categories: (1) outreach and education, (2) consultation, (3) cooperation, (4) collaboration, and (5) partnership^10^. Stakeholders classified the projects as the following: 25% outreach and education, 12% consultation, 15% cooperation, 27% collaboration, and 22% partnership.

**Table 5:** Follow-up Survey Results (n=20 Participants, 26 Projects)

Variable	Category	N (%)	
Did they watch information video	Yes	9 (45%)	
	No	10 (50%)	
	*Missing*	1 (5%)	
If so, did they find is useful (n=9)	Yes	8 (88.9%)	
	No	1 (11.1%)	
Where they able to understand results given	Yes	18 (90%)	
	No	1 (5%)	
	*Missing*	1 (5%)	
Did they feel project was correctly classified? (n=14)^1^	Yes	12 (85.7%)	
	No	1 (7.1%)	
	*Missing*	1 (7.1%)	
Did staff have to take off work or reduce schedule due to COVID-19	Yes	4 (20%)	
	No	14 (70%)	
	*Missing*	2 (10%)	
Partnership been impacted by COVID-19	A great deal	3 (15%)	
	Somewhat	7 (35%)	
	In a limited way	5 (25%)	
	Not at all	4 (20%)	
	*Missing*	1 (5%)	
		Median (IQR)	Range
How likely to recommend REST to a colleague^2^		8.0 (2.0)	6–10
Importance of measuring stakeholder engagement in research^3^		5.0 (1.0)	4–5
Feasibility of intervention measure^4^			
The Research Engagement Survey Tool (REST) seems implementable.		5.0 (1.0)	3–5
The REST seems possible.		4.0 (1.0)	3–5
The REST seems doable.		4.0 (1.0)	3–5
The REST seems easy to use.		4.0 (1.0)	3–5

^1^Only asked of projects with 5 of more stakeholders completing survey (n=14, 1 missing response)

^2^Measured on a scale of 0 (not at all likely) to 10 (extremely likely)

^3^Measured on a scale of 1 (not at all important) to 5 (extremely important)

^4^Scale ranges from 1 (completely disagree) to 5 (completely agree)

*IQR* interquartile range

## DISCUSSION

The purpose of this paper is to present the implementation and uptake of the Research Engagement Survey Tool (REST) among research teams. The data presented here indicate that REST implementation is feasible in a volunteer group of ongoing research projects. These projects were from multiple sources (e.g., PCORI, NIH), suggesting that the tool has some flexible appeal for many different types of research projects. Time and workload were perceived barriers to implementation of REST; however, stakeholder access to the Internet was not reported as a significant barrier.

Many project staff groups reported interest in using the tool, but less than half of the projects that originally agreed to use it did so. We do not know what interfered with their initial intention to use, and so perhaps they might in the future. Because some (69%) of the projects had direct contact with participants, we do not know how many received it or instructions on completions or the nature of the request to complete.

This type of implementation of a research engagement evaluation tool takes resources and time. However, the qualitative research methods often used to evaluate stakeholder engagement also requires resources and time. REST utilization of the type we evaluated might be necessary, but there are other ways to promote engagement and use of a tool like this one to measure the extent of engagement. One strategy would be for funders to require projects to carefully evaluate their engagement as part of their ongoing research and to offer this tool as an evaluation metric.

This project has limitations that constrain its generalizability. First, we only emailed to specific projects that were funded or otherwise associated with PCORI or NIH since this data is publicly available (convenience sample). There might be other ways of encouraging engagement and evaluation of the engagement activities in a broader set of projects, over time. We suggest that mailings be conducted with a larger group of research projects and groups, to let them know about the idea of using an evaluation tool like this one in their projects. We also hope that we will be able to show them how it could benefit their projects to use such a tool over time to gauge the strength of their engagement efforts. This project only used a single tool and timepoint to measure the strength of engagement in research, and using multiple tools at multiple timepoints, or perhaps a mixed methods approach might yield more nuanced results and findings. There is a bias in the reports of the ratings of the usefulness of the results that is not apparent from the study flow. Only 114 of the 482 (479 eligible) replied to the initial request and then only 86 completed the baseline—so 86 of 479 (18%) provided some data. We do not know anything about the non-responders, and this could form the focus of another study.

From this project, we learned overall that it is quite possible to implement the REST as an evaluation tool in large complex stakeholder-engaged research projects. Most of the project groups agreed with the ratings given by their completed survey data, suggesting that there is acceptability of the results with practitioners in the data provided by the REST. We do not fully know how the REST measures change over time, in response to research participation, and this is a topic for future research projects.

## Supplementary Information

Supplemental Figure 1. Consort Diagram of Implementation Study (DOCX 50 kb)
