# Supplementary material for: A Study Examining the Usefulness of a New Measure of Research Engagement
Source: J Gen Intern Med. 2022 Mar 29;37(Suppl 1):50–6. doi: 10.1007/s11606-021-06993-1 (PMC8960689; doi:10.1007/s11606-021-06993-1)
Supplement: Supplementary file 1 — Consort Diagram of Implementation Study (DOCX 50 kb) [file 11606_2021_6993_MOESM1_ESM.docx]

**Supplemental Figure 1. Consort Diagram of Implementation Study**

Assessed for eligibility (n=117)

Excluded (n=3)

- Not meeting inclusion criteria (n=3)

Completed Baseline Survey (n=86)

Consented (n=114)

## Enrollment

Indicated Willingness to Implement REST (n=47; n=41 teams)

Implemented REST (n=21; n=20 teams)

Completed Follow up Web Survey (n=20; n=20 teams)

Complete Zoom Interview (n=13; n=13 teams)

Not Willing to Implement REST (n=39)

Did not Implement REST (n=26, 21 teams)

Survey 2 (n=374)
